# *LPA* Gene Polymorphisms and Gene Expression Associated with Coronary Artery Disease

**DOI:** 10.1155/2017/4138376

**Published:** 2017-12-31

**Authors:** Zi-Kai Song, Hong-Yan Cao, Hai-Di Wu, Li-Ting Zhou, Ling Qin

**Affiliations:** ^1^Department of Cardiology, The First Hospital of Jilin University, Changchun, China; ^2^Department of Occupational and Environmental Health, School of Public Health, Jilin University, Changchun, China

## Abstract

The aim of our study was to investigate the influence of* LPA* gene polymorphisms for CAD risk and Lp(a) in a case-control study of Chinese Han population. In addition, we further analyzed the effect of* LPA* gene expression on plasma levels of Lp(a) and CAD risk. First, five SNPs (rs1367211, rs3127596, rs6415085, rs9347438, and rs9364559) in* LPA* gene were genotyped using the SEQUENOM Mass-ARRAY system in two groups. Second, we used quantitative real-time PCR to examine the mRNA expression levels of* LPA* gene in 92 cases and 32 controls. Results showed that the frequency of rs6415085-T allele was significantly higher in case group than that in control group (*P* < 0.05). Haplotypes were not associated with CAD risk (*P* > 0.05). And cases with the TT/TG genotype had significantly higher plasma Lp(a) levels compared with those that have the rs6415085 GG genotype (*P* < 0.05). Additionally, the mRNA expression levels in case group are significantly higher than that in control group (*P* < 0.05). Our study confirmed that rs6415085 was associated with CAD and increased plasma Lp(a) levels. And increased mRNA expression level of* LPA* gene may be a mechanism in development of CAD.

## 1. Introduction

The developing of CAD was associated with many risk factors in many studies [1, 2]. Among these factors, the lipid level is one of the main CAD risk factors. Therefore, total cholesterol, high-density lipoprotein cholesterol (HDL-C), and several other conventional risk factors are often used to predict the risk for cardiovascular disease (CVD) [3, 4]. In the study by Emerging Risk Factors Collaboration, for individuals without known CVD, the combination of lipoprotein(a), apolipoprotein B and A-I, or lipoprotein-associated phospholipase A2 mass to risk scores containing total cholesterol and HDL-C led to slight improvement in CVD prediction [5]. However, the mechanism of action of lipoprotein(a) [Lp(a)] in cardiovascular risk and the significance of treatment for Lp(a) are not clear.

The structure of Lp(a) is complex. It is a plasma lipoprotein consisting of a cholesterol-rich low-density lipoprotein cholesterol (LDL) particle with one molecule of apolipoprotein B100 and an additional protein, apolipoprotein(a), attached via a disulphide bond [6]. Additionally, the plasma levels of Lp(a) are effected by genetic factors. A Genome Wide Association Study (GWAS) studied by R. Clarke showed that* LPA* gene variants were strongly associated with both an increased plasma levels of Lp(a) and an increased risk of coronary disease [7].

*LPA*, located on chromosome 6q25-q26, encodes the apolipoprotein(a) component of the Lp(a) lipoprotein particle. In studies for the association between* LPA* and CAD risk and plasma Lp(a) levels, rs10455872 and rs3798220 were strongly associated with increased plasma levels of Lp(a), a reduced copy number in* LPA* (which determines the number of kringle IV-type 2 repeats), and a small Lp(a) lipoprotein size [7, 8]. However, another three loci (rs3127596, rs6415084, and rs9364559) were also significantly associated with CAD risk, and rs3127596 and rs6415084 were also significantly associated with increased plasma levels of Lp(a). Therefore, the aim of our study was to investigate a possible association between* LPA* gene polymorphisms and CAD risk in a case-control study of Chinese Han population. In addition, we further analyzed the effect of* LPA* gene expression on plasma Lp(a) levels and CAD risk in Chinese Han population.

## 2. Materials and Methods

### 2.1. Study Subjects

92 cases and 32 controls were collected for genetic and mRNA expression level analysis. All subjects were Chinese of Han descent from the First Hospital of Jilin University during February 2013 and March 2014. Diagnosis of CAD was carried independently by at least two well-trained physicians based on the following criteria. All patients were identified with CAD by coronary computed tomographic angiography (SIEMNS Somatom Definition AS + 128 row spiral CT). CAD was defined by ≥50% stenosis in any major coronary artery. Patients with nonatherosclerotic vascular diseases, congenital heart disease, cardiomyopathy, valvular disease, renal or hepatic disease, and cancer were excluded. All control subjects had ECG, chest X-ray, and serum analysis. They were classified as healthy subjects based on their normal physical examination results coupled with the absence of personal or family history and reasons for being suspected CAD.

The presence of cardiovascular risk factors, including diabetes mellitus (fasting blood glucose ≥ 7.0 mmol/L, and/or using glucose-lowering medication, including insulin), hypertension, and cigarette smoking, was obtained from all participants. Hypertension was defined according to seated blood pressure readings of 140/90 mmHg and higher, and/or subjects' receiving antihypertensive medication. In this study, a smoking habit was defined as a daily intake of >10 cigarettes [9].

This study complied with the Declaration of Helsinki. All the subjects have written informed consent for the study, which was approved by ethics committee of Jilin University, Changchun, China.

### 2.2. Laboratory Examination

Before starting the study, all participants underwent an initial screening assessment that included medical history, vital signs, a 12-lead electrocardiogram, and measurement of lipid variables and novel risk factors. Venous blood was collected in the morning after an overnight (8–12 hours) fast. Serum/plasma samples were frozen and stored at −80°C prior to analysis. All measurements were performed in a central laboratory.

### 2.3. SNP Selection, Identification, and Genotyping

The candidate single nucleotide polymorphisms (SNPs) (rs3127596 and rs9364559) were selected from a GWAS study. Another three tagging SNPs (rs1367211, rs6415085, and rs9347438) were chosen from genotyped SNPs in Chinese Han population (CHB) of the HapMap project (the Phase I database). All SNPs were restricted to minor allele frequency bigger than 15% in HAPMAP-CHB database (https://www.hapmap.org). Genomic DNA used for PCR amplification was extracted from the whole blood sample using a DNA extraction kit (Takara, China). Primers of amplification and extension were used by AssayDesigner3.1 software. Amplification and extension primers sequences of five loci in* LPA* gene were described by us previously [10]. Genotypes were assigned real-time using Typer 4.0 software (Sequenom). As quality controls, 5–10% of the samples were genotyped in duplicate. No inconsistencies were observed. Controls distributed in each 384-well plate were also consistent. Cluster plots were made of the signals from the low and the high mass allele.

### 2.4. RNA Isolation and Quantification

High-quality total RNA was isolated from human peripheral blood samples from case and control groups with different genotypes in Chinese Han population, and 500 ng of total RNA was reverse transcribed. LPA mRNAs were quantified by real-time quantitative polymerase chain reaction using the 5′ nuclease assay and the ABI Prism 7300 Sequence Detection System (Applied Biosystems, Foster City, CA). Oligonucleotide primers and TaqMan probes are given: 5′-GGGACAAATAAATGGGCAGGT-3′, 5′-AATGAAGAGGATGCACAGAGAGG3′. The relative gene expression levels were calculated by 2^−  ΔCt^(ΔCt = Ct_target_–Ct_actin_) [11].

### 2.5. Statistical Analysis

Data were expressed as percentages of total for categorical variables, or mean ± SD. The statistical analyses on the characteristics of the subjects were performed with Pearson *χ*^2^ test for the categorical variables such as sex, smokers, drinkers, hypertension, and diabetes and with Student's *t*-test for the continuous variable of age, total cholesterol (TC), triglyceride (TG), LDL-C, and HDL-C with normal distribution. Association between different groups and alleles or genotypes of* LPA* gene polymorphisms was analyzed using either *χ*^2^ or Fisher exact test. Plasma Lp(a) levels and mRNA expression levels of the* LPA* gene were nonnormal distribution data, which were expressed as M ± Q. For the association between SNPs and the mRNA expression level of the* LPA* gene, rank-sum test was used. SPSS 16.0 was used for above analyses.

## 3. Results

### 3.1. Characteristics of Participants

The baseline characteristics of case and control groups are shown in Table 1. There was no significant difference of the mean age, sex, rate of drinking, prevalence of diabetes mellitus, body mass index (BMI), serum TG, and TC level between two groups. Compared with control group, case group had more smokers and more individuals with hypertension. Additionally, compared with control group, case group had higher level of serum LDL-C, HDL-C and Lp(a) (*P* < 0.05).

### 3.2. Allele and Genotype Analysis

The distributions of alleles and genotypes of five loci among participants were presented in Table 2. Analysis with SPSS 16.0 showed that allele frequencies of rs6415085 were significantly higher in case group than that in control group (*P* < 0.05). But, there was no difference of genotype frequencies for rs6415085 between two groups (*P* > 0.05). Additionally, there were no differences in genotype and allele frequencies for the other four SNPs between two groups (*P* > 0.05).

### 3.3. Linkage Disequilibrium (LD) Analysis

**Figure 1:**
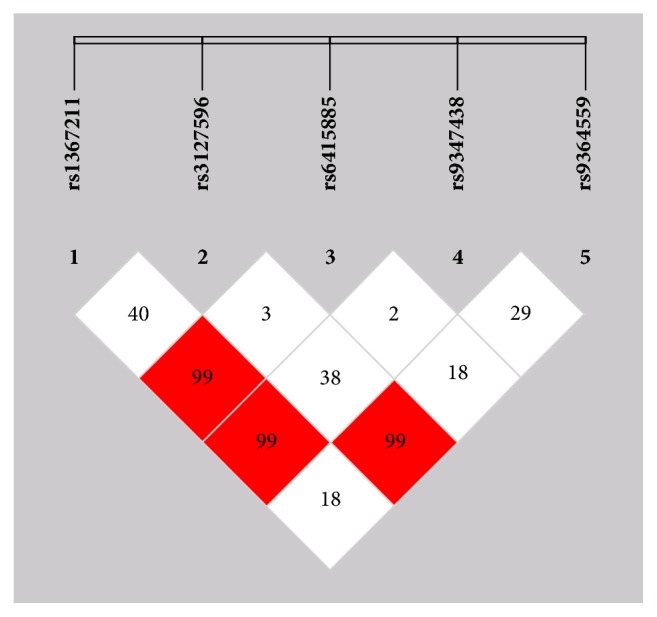
Linkage disequilibrium (LD) pattern between the five LPA single nucleotide polymorphisms (SNPs) genotyped in this study. Rs1367211 and rs6415085, rs1367211 and rs9347438, and rs3127596 and rs9364559 were in tight LD (*D*′ = 0.99) and were organized in a single haplotype block.

LD analysis for five SNPs on* LPA* gene in cases and controls, respectively, results showed that rs1367211 and rs6415085 (*D*′ = 0.99), rs1367211 and rs9347438 (*D*′ = 0.99), and rs3127596 and rs9364559 (*D*′ = 0.99) were in tight LD (Figure 1). Results of haplotype analysis showed that haplotypes were not associated with CAD risk (*P* > 0.05).

### 3.4. The Effect of* LPA* SNPs on Serum Lp(a) Levels in Chinese Han Population

Each SNP was tested for an association with Lp(a) levels. Results from this analysis are presented in Table 3. There were no significant differences in plasma Lp(a) levels between case and control groups with different genotypes (*P* > 0.05). But, we further analyzed the effect of* LPA* SNPs on serum Lp(a) levels in cases and controls, respectively. Results showed that patients with the TT/TG genotype had significantly higher plasma Lp(a) levels compared with those that have the rs6415085 GG genotype (*P* < 0.05).

### 3.5. Real-Time RT-PCR Analyses Identified High Expression Level of* LPA* Gene Related to CAD Risk

**Figure 2:**
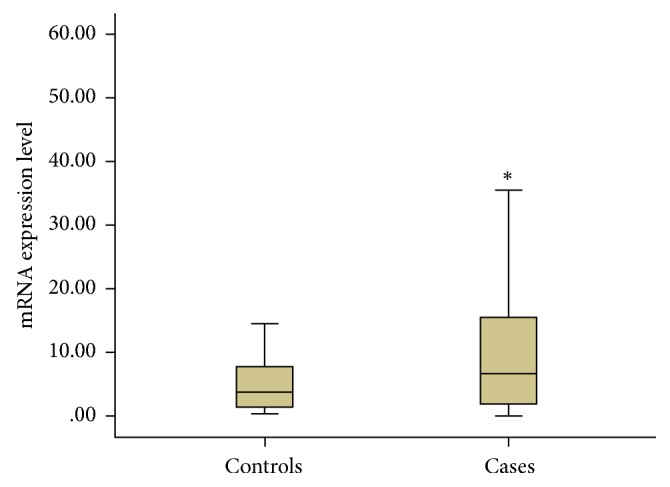
The mRNA expression levels of LPA gene in case and control groups (M ± Q).^*∗*^ after adjustment of the presence of smoking, hypertension by logistic regression analysis, *P* < 0.05, compared with controls.

Further comparing the mRNA expression levels of* LPA* gene in two groups (Figure 2), the mRNA expression levels in case group are significantly higher than that in control group (*P* < 0.05).

### 3.6. Assessment of Association between SNPs and the mRNA Expression Level of* LPA* Gene

We used quantitative real-time polymerase chain reaction (qRT-PCR) analysis to assess whether SNPs were associated with the expression level of* LPA* gene. The data showed that there was no significant association between SNPs and the expression level of* LPA* gene (*P* > 0.05) (Table 4).

### 3.7. Logistic Regression Analysis

As shown in Table 5, ageing, sex, diabetes mellitus, hypertension, smoking, drinking, TC, TG, LDL-C, HDL-C, Lp(a), mRNA expression level of* LPA* gene, and five SNPs were included in the multivariate logistic regression model. Result showed that LDL-C, smoking, Lp(a), and high mRNA expression level of* LPA* gene all increased the risk of CAD. But LDL-C was the most predictive risk factor for CAD.

## 4. Discussion

The pathogenesis of CAD is not clear. But many studies proved that the interaction of genetic and environmental factors is the main factor for the developing of CAD. In addition to environmental factors such as age, gender, bad behavior, poor diet, obesity, dyslipidemia, hypertension, and diabetes, GWAS study has identified several novel susceptibility loci for CAD [12–16]. But many SNPs appear in noncoding regions of the genome. Therefore, the complex mechanisms are still largely unknown. Many studies have confirmed that SNPs which influence mRNA expression are known as expression Quantitative Trait Loci (eQTL) [17–20].

In this study, we observed frequency of T allele of rs6415085 was increased in individuals with CAD. But there was no difference of genotype frequencies for rs6415085 between two groups. Further results of LD analysis showed that rs1367211 and rs6415085, rs1367211 and rs9347438, and rs3127596 and rs9364559 were in tight LD. But haplotypes were not associated with CAD risk. In addition, patients with the rs6415085 TT/TG genotype had significantly higher Lp(a) levels than that with the rs6415085 GG genotype. Therefore, above result suggests that* LPA* gene may be the potential mechanism leading to atherosclerosis and associated with an increased level of Lp(a). Above results are consistent with previous studies [21–23]. Rs10455872 and rs3798220 in* LPA* gene were proved strongly associated with plasma Lp(a) levels and CAD and reduce the copy number at* LPA* (which determines the number of kringle IV-type 2 repeats) and a small Lp(a) lipoprotein size [7, 8].

Meanwhile, we found that* LPA* gene expression was significantly increased in individuals with CAD compared to those without CAD. And CAD patients have higher plasma Lp(a) levels than healthy individuals. Therefore, these results further confirmed the relationship between Lp(a) levels and CAD risk [24–26].

The mechanism research by Niccoli et al. showed that higher plasma Lp(a) levels exhibited a higher prevalence of lipidic plaque at the site of the culprit stenosis, a wider lipid arc, and a higher prevalence of thin-cap fibroatheroma [27]. Therefore, the role of therapies targeting Lp(a) can decrease CAD risk through reduction of vulnerable plaque features of patients with CAD. Regression analysis results showed that the plasma Lp(a) and LDL-C levels are associated with CAD. Combined with above results, we speculate rs6415085 TT/TG genotype may, through increased plasma Lp(a) levels, increase CAD risk.

But our results showed that LPA mRNA expression level is not significantly increased in patients with rs6415085 TT/TG genotype. The above results are mainly due to rs6415085 being intron variant in noncoding regions of* LPA* gene. In the future through the study of introns and siRNA research it is expected to clear the mechanism of SNP.

A potential limitation of our study was the numbers of subject in our study, which may limit statistical power and generalization to the overall Chinese population. Nevertheless, our results are in accordance with former studies that suggested a putative influence of* LPA* gene polymorphisms in the mechanisms associated with CAD. In addition, we did not collect data of treatment for hyperlipidemia, which maybe affect the relationship between genotypes and lipid levels. In further study, we should collect more comprehensive data about angiography and history of treatment to analyze association between gene variants, lipid level, and CAD severity.

In conclusion, our study confirmed that T allele of rs6415085 was associated with CAD and rs6415085 TT/TG genotype which maybe increased plasma Lp(a) levels. And increased mRNA expression level of* LPA* gene may be a mechanism in development of CAD. However, further studies encompassing larger number of patients are needed to confirm the results obtained in this study.

## Figures and Tables

**Table 1:** Base characteristics of case and control group.

Variable	Case group (*n* = 92)	Control group (*n* = 32)	*P*
Age^a^ (year)	66.34 ± 11.83	62.18 ± 11.07	0.296
Sex (%)	58.7	75.0	0.100
BMI^a^ (kg/m^2^)	24.19 ± 2.68	24.01 ± 3.31	0.431
Smoking (%)	37.5	18.1	0.021
Drinking (%)	20.0	19.2	0.842
Hypertension (%)	49.4	28.1	0.037
Diabetes mellitus (%)	25.8	21.9	0.656
TC^a^ (mmol/L)	5.09 ± 0.88	4.77 ± 1.07	0.143
TG^a^ (mmol/L)	2.26 ± 1.39	1.77 ± 1.01	0.074
LDL-C^a^ (mmol/L)	3.08 ± 0.84	2.74 ± 0.82	0.021
HDL-C^a^ (mmol/L)	1.21 ± 0.32	1.16 ± 0.24	0.032
Lp(a)^b^ (mmol/L)	226.34 ± 123.25	195.96 ± 117.19	0.000

^a^Variables are described based on mean ± SD. ^b^Variables are described based on median ± QR.

**Table 2:** SNPs loci genotype and allelic frequency distribution of *LPA* gene in two groups.

SNPs	Genotype						Allelic			
	Cases (92)			Controls (32)			Cases (92)		Controls (32)	
Rs1367211	2	27	58	3	12	17	31	143	18	46
Rs3127596	63	22	3	27	4	0	148	28	58	4
Rs9364559	40	40	12	11	18	2	120	64	40	22
Rs6415085	54	17	15	23	2	3	125	47^*∗*^	48	8
Rs9347438	13	43	31	1	16	14	69	105	18	44

^*∗*^ *P* < 0.05.

**Table 3:** The association between SNPs and serum Lp(a) level (M ± Q).

SNPs	Case group	Control group	*χ* ^2^	*P*
rs1367211				
AA + AG	241.00 ± 75.91	216.24 ± 97.19	1.915	0.166
GG	237.67 ± 172.35	195.96 ± 84.54		
rs3127596				
GG + GA	242.23 ± 104.12	195.42 ± 87.46	1.077	0.299
AA	218.82 ± 157.81	171.94 ± 91.94		
rs9364559				
GG + GA	246.57 ± 129.56	211.94 ± 78.15	0.893	0.345
AA	258.04 ± 193.16	202.23 ± 91.21		
rs6415085				
TT + TG	279.50 ± 95.04^*∗*^	218.45 ± 107.26	2.234	0.135
GG	197.56 ± 99.74	194.24 ± 81.11		
rs9347438				
CC + CT	226.56 ± 120.57	188.00 ± 123.85	1.568	0.211
TT	238.82 ± 175.95	194.24 ± 93.28		

^*∗*^ *P* < 0.05.

**Table 4:** The association between SNPs and the mRNA expression level of LPA gene (M ± Q).

SNPs	Case group	Control group	*χ* ^2^	*P*
rs1367211				
AA + AG	4.955 × 10^−2^ ± 14.208 × 10^−2^	3.874 × 10^−2^ ± 6.870 × 10^−2^	1.401	0.237
rs3127596				
GG + GA	8.133 × 10^−2^ ± 31.607 × 10^−2^	5.833 × 10^−2^ ± 2.956 × 10^−2^	1.293	0.256
rs9364559				
GG + GA	6.629 × 10^−2^ ± 28.219 × 10^−2^	5.834 × 10^−2^ ± 7.659 × 10^−2^	0.872	0.350
rs6415085				
TT + TG	4.374 × 10^−2^ ± 30.729 × 10^−2^	9.554 × 10^−2^ ± 40.637 × 10^−2^	0.195	0.659
rs9347438				
CC + CT	8.597 × 10^−2^ ± 32.258 × 10^−2^	5.555 × 10^−2^ ± 8.500 × 10^−2^	3.622	0.057

**Table 5:** Multivariate logistic regression analysis showing the predictors of CAD.

Risk factor	B	S.E.	Wald	*P*
Lp(a)	0.010	0.004	7.068	0.008
LDL-C	1.438	0.483	8.852	0.003
Smoking	−1.214	0.607	3.997	0.046
LPA expression level	0.093	0.042	4.833	0.028
